# Postprandial low-grade inflammation does not specifically require TLR4 activation in the rat

**DOI:** 10.1186/s12986-017-0220-4

**Published:** 2017-10-19

**Authors:** Dominique Hermier, Véronique Mathé, Annaïg Lan, Clélia Santini, Annie Quignard-Boulangé, Jean-François Huneau, François Mariotti

**Affiliations:** UMR Physiologie de la Nutrition et du Comportement Alimentaire, AgroParisTech, INRA, Université Paris-Saclay, 16 rue Claude Bernard, F-75005 Paris, France

**Keywords:** Postprandial, Inflammation, TLR, Adipose tissue

## Abstract

**Background:**

Toll-like receptor 4 (TLR4), an innate immune receptor, is suspected to play a key role in the postprandial inflammation that is induced by a high-fat meal rich in saturated fatty acids (SFA). Our objective was to test this hypothesis by using a specific competitive inhibitor of TLR4 (INH) vs vehicle (VEH) administered immediately before a high-SFA meal in rats.

**Methods:**

First, in a cross-over kinetic study of 12 rats receiving INH and VEH *i.v.* 10 min before the test meal, we measured plasma inflammatory and vascular markers for 6 h. Then, in 20 rats, 3 h after INH or VEH followed by the test meal (parallel study), we measured the mRNA level of a set of cytokines (*Il1-β*, *Il-6*, *Tnfα*, *Mcp-1*, *Pai-1*), and of *Tlr4* and *Tlr2* in the adipose tissue and the liver, and that of adhesion molecules (*Icam-1* and *Vcam-1*) in the aorta.

**Results:**

Plasma IL-6 and PAI-1 increased >4-fold at 3–4 h after test-meals, very similarly after INH as compared to VEH. The expression of TLR2 and of all measured cytokine genes in the adipose tissue was dramatically higher after INH (vs VEH). In the liver, gene expression of *Il1-β*, *Tnfα*, *Mcp-1* and *Tlr2*, was also higher after INH, though more moderately, whereas that of *Il-6* and *Pai-1* was similar between groups. INH did not affect mRNA level of *Icam-1* and *Vcam-1* in the aorta.

**Conclusion:**

TLR4 activation is not specifically required to mediate systemic postprandial inflammation and we propose that TLR2 and TLR4 exert a dual and interdependent mediation of the postprandial inflammatory response, at least in the adipose tissue.

## Background

Epidemiological and experimental data indicate that diets rich in saturated fatty acids (SFA) and simple sugars favour the development of metabolic and physiological dysregulations that characterize the metabolic syndrome and increase the risk of cardiovascular disease and type-2 diabetes [1]. These dysregulations includes oxidative stress, low-grade inflammation and endothelial dysfunction, which are well known as being pivotal to the initiation and progression of the metabolic syndrome. Importantly, in the last decade, a large body of evidence has accumulated demonstrating that a single high-lipid/high-sucrose meal transiently elicits a cluster of low-grade postprandial inflammatory responses (PPIR) [2–5]. PPIR can be measured in plasma as an increased concentration in a number of inflammatory and vascular markers. This plasma increase has been reported in response to a high-fat challenge in healthy human subjects [6, 7] as well as in the rat [3, 8].

The occurrence of PPIR is very relevant to the nutritional pathophysiology of the metabolic syndrome. Indeed, PPIR is an acute response to the allostatic stress evoked by an excess of dietary fats (especially saturated ones) and simple sugars, and is augmented in individuals with risk factors of CVD and type-II diabetes [2, 9, 10]. The present paradigm is that the repetition of a high magnitude PPIR, although silent and transient, is the mechanism that mediates the effect of diet or specific nutrients on the initiation and progression of the cardiometabolic risk.

Characterization of PPIR has been largely improved recently, however underlying molecular mechanisms remain poorly understood. The plasma rise in glucose and triglyceride (in the form of chylomicrons and their remnants, essentially) activates inflammatory signalling pathways in leukocytes, vascular endothelium, and visceral adipose tissue [4, 11, 12]. The inflammatory signalling pathways involved during PPIR remain a matter of debate, however postprandial activation of the Toll-like receptors 2/4 (TLR 2/4)/NF-κB pathway in the adipose tissue is a promising hypothesis. Indeed the NF-κB p65 expression in the human [13], or translocation in the rat [3] are triggered by a high-fat meal (HFM) rich in SFA in the adipose tissue. However, the level of the contribution of the TLR to PPIR remains to be determined. The objective of the study is to determine whether or not, and to what extent, the onset of PPIR is mediated by the activation of TLR4.

For this purpose, the rat is a very good model, since it has been shown to display a PPIR resembling that described in humans in response to a HFM [3, 8, 14]. Furthermore, the rat is sensitive to a long-term pro-inflammatory allostatic stress inasmuch as rats given a diet whose composition is similar to that of the HFM develop a metabolic syndrome with fasting elevation in plasma and tissue pro-inflammatory markers [3, 14–16]. Finally, pre-clinical studies in this species showed that TLR4 activation was inhibited by Eritoran (E5564), a specific competitive TLR4 antagonist [17]. In the present work, the implication of TLR4 in the occurrence of the PPIR was first determined by characterizing the PPIR kinetics in plasma after a single HFM in rats given Eritoran as a TLR4 inhibitor (INH) or its vehicle (VEH) as a control, in a cross-over design. This study was also used to determine the most appropriate time for further analysis of the PPIR in the tissues, and its modulation by TLR4 inactivation. Therefore, in the second study, rats were killed at that time (i.e. 3 h) after a HFM with Eritoran or the vehicle in a parallel design. Despite that all fatty acids may trigger a PPIR when provided in large amounts in a HFM [18], we used a meal rich in SFA as the classical model for PPIR study. Excessive SFA intake, such as found in typical western diets, have also been reported to favour chronic low-grade inflammation in association with TLR activation [19].

## Methods

### Animals and treatments

Male Wistar rats (breed RccHanWIST, Envigo, Gannat, France), initially weighing 250–274 g (11 wk), were collectively housed (3–4 rats/cage on wired floor + wood litter) in a room maintained at 22 ± 1 °C with an artificial 12:12 h light–dark cycle (lights on at 06:00 a.m.). They were fed ad libitum a standard pelleted diet (SAFE A04, Augy, France) and acclimated to local conditions for 1 week, during which body weight was monitored.

Eritoran (E5564) and the vehicule were a gift of Esai Inc. (Andover, MA, USA). They were provided as powder in vials (7.46 mg Eritoran per vial, as Eritoran tetrasodium salt, corresponding to 7 mg Eritoran). One mL sterile water was added to each vial prior injection. Eritoran dosage was 25 mg/kg body weight (as the maximal dose that has been used to inhibit TLR4 activation without adverse effects according to the manufacturer).

The HFM consisted in a 5-mL emulsion made of palm oil (1.1 g, 47% SFA -40% C16:0 and 4% C18:0-, 43% monounsaturated FA -38% C18:1 n-9-, 10% n-6 polyunsaturated FA and 0.23% n-3 polyunsaturated FA), sucrose (0.88 g), milk protein (0.88 g) and water (2.1 g). It provided 18.3 kcal (60% as lipid, 20% as carbohydrate and 20% as protein).

### Postprandial kinetics

#### Study design

Twelve rats were used in a cross-over design. The rats were studied in two explorations after a HFM after receiving Eritoran (INH) or its vehicle (VEH), in randomized order, separated by one-week recovery. They weighed 296 ± 10 g (12 wk) at the first exploration and 328 ± 19 g (13 wk) at the second one. At the second exploration, the same 12 rats entered into the same procedure, but received the treatment alternative to the first exploration. Each exploration was as follows. After having been fasted overnight, an indwelling catheter, filled with heparinized 2% NaCl (50 U/mL), was inserted and secured into a lateral tail vein to allow repeated blood sampling with minimal discomfort for the conscious animal. Venous blood (300 μL) was sampled in fasted rats (T0) then INH (7 mg) or VEH was injected slowly via the catheter. Ten minutes later, the HFM (5 mL) was administered by gavage. Venous blood (300 μL) was then sampled 1, 2, 3, 4 and 6 h after the HFM. After each blood sampling, 300 μL NaCl 0.9% were injected via the catheter to maintain blood volume. Blood samples were drawn into EDTA prechilled tubes and were centrifuged for 10 min at 2000*g at 4 °C. Plasma was stored at −20 °C for further determinations (as described below).

#### Biochemical analyses

Glucose concentration was determined immediately on whole blood taken from conscious rats with an Accu-Check® glucometer (Roche Diagnostics, Meylan, France). Plasma triglyceride concentration was determined by a colorimetric enzymatic method using the kit provided by Randox (Roissy, France) [20]. Plasma concentrations of inflammatory and vascular markers were determined simultaneously by multiplex immunoassay on a Luminex-200 analyser (Biorad, Hercules, CA, USA), using the Milliplex Rat Adipokine Panel for interleukin 6 (IL-6), plasminogen activator inhibitor-1 (PAI-1), interleukin 1β (IL-1β) and monocyte chemoattractant protein-1 (MCP-1) and the Milliplex Rat Cardiovascular Panel 2 for intercellular adhesion molecule 1 (ICAM-1) and E-selectin (Millipore, Molsheim, France).

### Postprandial tissue inflammation

#### Study design

Twenty rats (307 ± 11 g, 12 wk) were used in another study with a parallel design. In vivo procedures were the same as above, except that rats were allocated to only one treatment (INH or VEH, *n* = 10 in each group). Venous blood was sampled before and 3 h after the HFM. Three hours after meal was expected to be the peak of plasma IL-6 and PAI-1 increase, which was confirmed by the first study (see Results). A first sample (300 μL) was drawn into EDTA prechilled tubes and treated as described above (postprandial kinetics). Immediately after the 3 h blood sampling, rats were anaesthetized with isoflurane, then blood were taken from the caudal vena cava. Blood glucose concentration was determined as described above. Rats were killed by exsanguination, the liver and the epididymal adipose tissue were weighed, and a sample was snap-frozen in liquid nitrogen, then stored at −80 °C until gene expression analysis. The thoracic aorta was quickly dissected out and washed, and the inner part (corresponding to the intima) was collected by delicate rubbing with a scalpel.

#### Analyses

Real-time quantitative PCR (RT-qPCR). Total RNA was extracted using Trizol reagent from adipose tissue, liver and aorta samples. Five hundred nanograms of total RNA were converted into cDNA using the High Capacity cDNA Reverse-Transcription Kit (Applied Biosystems, Villebon-sur-Yvette, France) on a PTC-200 thermocycler (MJ Research/Biorad). Real-time PCR amplifications were performed with a Prism7300 sequence detection system using SYBR Green MasterMix (Applied Biosystems). *Il-6*, *Il-1β*, *Il-10 (Interleukin-10)*, *Tnf-α (Tumor necrosis factor-α)*, *Pai-1*, *Mcp-1*, *Tlr2*, *Tlr4*, *Fas (Fatty acid synthase)*, *Lpl (Lipoprotein lipase)*, *Icam-1* and *Vcam-1* (*Vascular cell adhesion molecule-1*) mRNA levels were expressed as a ratio of *Hprt1* mRNA levels. Primer sequences (Table 1) were designed using Primer Express™ (Applied Biosystems) software and were from Eurogentec (Seraing, Belgium). Gene expression was determined using the 2^-ΔCt^ formula using *Hprt1* as the reference (ΔCt = Ct target gene – Ct *Hprt1*).

**Table 1:** Primer sequences used in the quantitative RT-qPCR analysis

Gene	Forward sequence	Reverse sequence
*Il-6*	AAGTCGGAGGCTTAATTACATATGTTC	TCATCGCTGTTCATACAATCAGAA
*Il-1β*	GCACCTTCTTTTCCTTCATC	GCCGTCTTTCATCACACA
*Tnfα*	TGTCTTTGAGATCCATGCCATT	TCGTAGCAAACCACCAAGCA
*Il-10*	CGGGGTGACAATAACTGC	CCTGGGGCATCACTTCTAC
*Pai-1*	GATCTTGACCTTTTGTAGTGCTTGTG	TGGGCATGACTGACATCTTCA
*Mcp-1*	GGCTCAGCCAGATGCAGTTAA	CCAGCCTACTCATTGGGATCA
*Tlr4*	CTGTTGGATGGAAAAGCC	GAGGTCGTTGAGGTTAGAAGC
*Tlr2*	GGGACCTTTGCTATGATGC	CTGTTTTGTGGCTCTTTTCG
*Lpl*	GGACTGAGGATGGCAAGCA	GGCAGGGTGAAGGGAATGTT
*Fas*	TGC-TCC-CAG-CTG-CAG-GC	GCC-CGG-TAG-CTC-TGG-GTG-TA
*Icam-1*	GAAGACAGCAGACCACTGTGCTT	CTCTGGGAACGAATACACAGTGAT
*Vcam-1*	TGT-GCC-TTG-CGG-ATG-GT	GAA-GTG-TGC-CCG-AAA-TAT-GGA
*Hprt*	CTCATGGACTGATTATGGACAGGAC	GCAGGTCAGCAAAGAACTTATAGCC

*Il-6* interleukin 6, *Il-1β* interleukin 1β, *Tnf-α* tumor necrosis factor α, *Il-10 interleukin 10, Pai-1* plasminogen activator inhibitor 1, *Mcp-1* monocyte chemoattractant protein 1, *Tlr* Toll-like receptor*, Lpl* Lipoprotein lipase*, Fas* Fatty acid synthase*, Icam-1* intercellular adhesion molecule 1, *Vcam-1* Vascular cell adhesion molecule 1, *Hprt* hypoxanthine-guanine phosphoribosyl transferase

### Statistical analyses

Data are reported as means ± SEs. Statistical analyses were performed with SAS (version 9.3; SAS Institute). Significance was set at *P* < 0.05. Kinetics data (from study #1) were analysed using mixed models (PROC MIXED) for repeated measurements with covariance structure modelling. Outcomes were explained by the following fixed factors: time after meal (pre/postprandial, i.e. 0 h, 1 h, 2 h, 3 h, 4 h, 5 h and 6 h), treatment (INH or VEH), period of treatment (first or second), treatment by time, and treatment by period interactions (to test for the possible existence of a carry-over). Treatment and time were considered as repeated factors on subject. Subject was analysed as a random effect. Multiple comparisons between treatment and time points were adjusted by Tukey-Kramer test. Data from study #2 were analysed with Student’s t tests.

## Results

### Postprandial kinetics

**Fig. 1:**
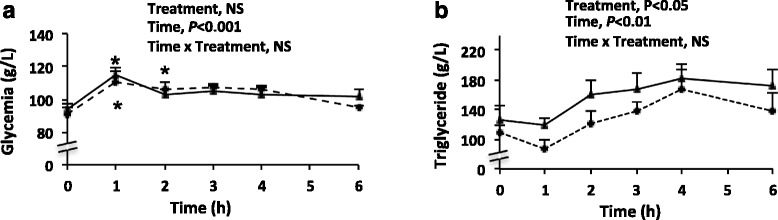
Kinetics of plasma glucose and triglyceride after a high-fat meal. Solid line, VEH treatment; dashed line, INH treatment; *, different from fasting value at *P* < 0.05; *n* = 12 per treatment

Glycemia averaged 0.90–0.95 g/L in fasting rats, reached a peak (1.10–1.15 g/L) 1 h after the HFM and then decreased progressively to fasting values (±1 g/L at T6) (Fig. 1a). Triglyceridemia averaged 1.10–1.30 g/L in fasting rats, then increased progressively to plateau at 4 h after the HFM (±1.70–1.80 g/L) (Fig. 1b). Time effect was significant for both parameters, and a significant overall treatment effect was observed for plasma triglyceride concentration, which may relate to the slightly higher fasting value before VEH, since we found no evidence for an effect of the treatment on the kinetics (i.e. a treatment by time effect). Furthermore, we found no effect of treatment (INH or VEH) on either glycemia or triglyceridemia at any time.

**Fig. 2:**
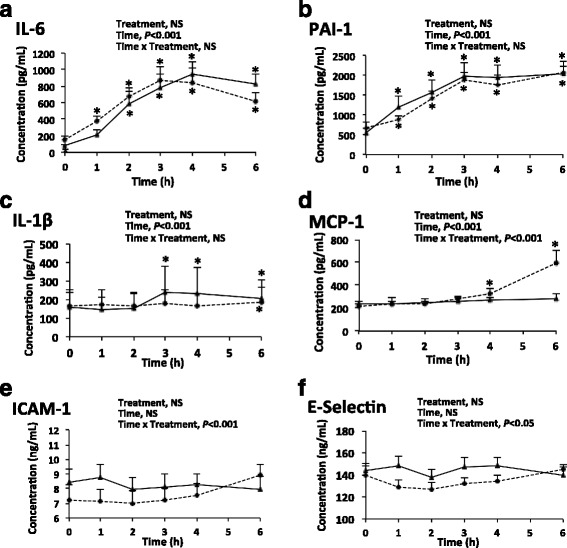
Kinetics of plasma inflammatory markers after a high-fat meal. Solid line, VEH group; dashed line, INH group; *, different from fasting value at *P* < 0.05; *n* = 6 per group

Plasma concentrations of IL-6 and PAI-1 increased progressively, reached a maximum 3–4 h after the HFM (with values 4–5-fold higher than in fasted rats), then plateaued (PAI-1) (Fig. 2b) or slightly decreased without returning to the fasting level (IL-6) (Fig. 2a). Plasma concentration of IL-1β slightly increased at T3 then plateaued in the VEH group, and did not vary in the INH group (Fig. 2c), whereas that of MCP-1 increased 4 h after the HFM in the INH group, and did not vary in the VEH group (Fig. 2d). Plasma concentration of ICAM-1 and E-selectin did not vary with time in response to the HFM (Fig. 2e and f). There was no effect of the treatment for any of the parameters.

In conclusion, only IL-6 and PAI-1 plasma concentrations increased markedly in response to the HFM, and reached their maxima after 3–4 h. Thus, the duration of 3 h after the HFM was chosen for a more detailed investigation of postprandial low-grade inflammation in various tissues.

### Tissue low-grade inflammation after a HFM

Glycemia averaged 0.95 g/L in fasted rats and rose to 1.15–1.20 g/L 3 h after the HFM, without difference between the INH and VEH groups at both times (data not shown).

In the adipose tissue, 3 h after the HFM, mRNA level of all inflammatory and vascular markers were significantly higher in the INH group, as well as mRNA level of *Tlr2* (Table 2). In contrast, *Tlr4* mRNA level did not differ between groups (Table 2). Besides, mRNA levels of two constitutive genes of the adipose tissue, i.e. *Fas* and *Lpl*, were not affected by the treatment (data not shown). In the liver, mRNA levels of the inflammatory and vascular markers and *Tlr2* were markedly lower than in the adipose tissue, excepted for *Il-1β.* These mRNA levels were still higher in the INH group, but to a lesser extent than in the adipose tissue, the differences being not significant for *Il-6* and *Pai-1*. Again, the level of *Tlr4* transcript did not differ between groups. In the aorta, there was no difference in mRNA level between groups for both *Icam-1* and *Vcam-1*.

**Table 2:** mRNA levels of inflammatory and vascular markers in the adipose tissue, the liver and the aorta 3 h after the high-fat meal

		VEH	INH	*P*
Adipose tissue	*IL-1β*	12 ± 2	135 ± 22	1.75389E-05
	*IL-6*	0.99 ± 0.23	57 ± 22	0.0223
	*IL-10*	8 ± 2	4.52 ± 1.32	0.0369
	*TNF-α*	12 ± 1	45 ± 4	2.49E-09
	*PAI-1*	227 ± 29	2.13 ± 0.38	0.0097
	*MCP-1*	127 ± 22	24.4 ± 5.6	0.0010
	*TLR4*	286 ± 24	0.87 ± 0.08	0.2915
	*TLR2*	438 ± 24	8.51 ± 2.42	9.77E-06
Liver	*IL-1β*	40 ± 5	2.93 ± 0.25	3.75E-06
	*IL-6*	0.129 ± 0.038	1.69 ± 0.36	0.1671
	*TNF-α*	3.27 ± 0.30	2.98 ± 0.40	0.0002
	*PAI-1*	6.63 ± 0.62	1.24 ± 0.13	0.1558
	*MCP-1*	5.89 ± 0.66	2.63 ± 0.87	8.09E-05
	*TLR4*	44.7 ± 3.89	0.83 ± 0.06	0.1284
	*TLR2*	22.6 ± 2.2	1.76 ± 0.17	0.0010
Aorta	*ICAM-1*	177 ± 24	0.98 ± 0.19	0.9460
	*VCAM-1*	1341 = 235	0.97 ± 0.12	0.9120

Values are means ± SEMs for 9–10 rats in each group. They are expressed as arbitrary units (2^-ΔCt^ formula using *Hprt1* as the reference, with ΔCt = Ct target gene – Ct *Hprt1*) for the VEH group, and as fold changes for the INH group. VEH, rats treated by the vehicle; INH, rats treated by the TLR4 competitive inhibitor, Eritoran

## Discussion

PPIR is a cluster of markers of a low-grade transient inflammation, which shares many features with low-grade chronic inflammation related to diet: a prominent role of dietary simple sugars and SFA as key initiators of the inflammatory response; a multiplicity of target-tissues, with an important contribution of the adipose tissue; assessment of inflammation magnitude by using some plasma inflammatory markers [4, 11, 12]. However, contrary to chronic low-grade inflammation, there is paucity of data regarding the signalling pathways involved at the molecular level. In particular, the activation of the TLR4/NF-κB pathway during PPIR has been proposed as a major determinant of PPIR in a few studies [3, 13], but the contribution of this pathway to the magnitude of PPIR has never been directly studied.

In the present study, Eritoran (E5564), a specific competitive TLR4 antagonist [17] was used to test this hypothesis in a rat model of PPIR [3].

Increase in plasma glucose and triglyceride concentrations were in line with the composition of the test-meal, the persistence of a high triglyceride up to 6 h being a consequence of the high lipid content of the meal (Fig. 1). Plasma PPIR features were variable (Fig. 2). Indeed, in the placebo group, the marked postprandial increase in IL-6 and PAI-1, the limited increase in IL-1β, as well as the absence of postprandial changes in ICAM were consistent with previous findings in the rat [3, 8] and with some human studies [6, 7]. As expected, the TLR4 antagonist (INH group) did not modify the postprandial handling of dietary lipids and carbohydrates as shown by plasma triglycerides and glucose. In contrast, one major but unexpected finding of this study was that plasma kinetics of PPIR markers in rats were not modified by TLR4 inhibition. There may be three optional explanations for such a finding: either TLR4 is not involved in PPIR, or another signalling pathway was activated concurrently to the inhibition of TLR4 signalling, or the increase in plasma IL-6 and PAI-1 is an experimental artefact.

This latter possibility was raised after some human studies found a similar increase in plasma IL-6 concentration after a HFM and after water. This non-physiological inflammatory response was shown to result from the use of an indwelling intravenous catheter [21, 22]. Although we also used an indwelling intravenous catheter, the hypothesis of an artefactual rise in plasma PPIR markers can be excluded under our experimental conditions, since we showed in a previous study using exactly the same experimental protocol that, contrary to a HFM, there was no change at all in plasma markers in a sham condition, i.e. after a water load, as assessed in a cross-over design study [3].

There is no doubt that a high-fat challenge triggers a rapid increase inflammatory response at the gene level in plasma mononuclear blood cells (PMBCs) in the human [7, 23]. In contrast, in the human adipose tissue, there is no clear-cut evidence of a postprandial increase in inflammatory genes, because of a possible confounding effect of repeated biopsies [13, 24–26]. Yet, a transcriptomic approach showed strong similarities in the activation of postprandial inflammatory pathways in PMBCs and white adipose tissue [27]. In the rodent however, plasma PPIR after a meal rich in SFA was paralleled by an increase of mRNA level of *Il-6* and *Il-1β* in the adipose tissue in the rat [3]. In the present study, mRNA level of inflammatory and vascular markers was dramatically higher after TLR4 inhibition than in the control group in the adipose tissue and, to a lesser extent, in the liver (Table 2). This finding raises the possibility that an alternative inflammatory pathway was activated in response to TLR4 inhibition during the postprandial period.

In our previous study in the rat, NF-κB was activated in the visceral the adipose tissue after a HFM, strongly suggesting the contribution of this inflammatory pathway to PPIR [3]. Not only TLR4, but also TLR2 triggers the NF-κB pathway, and they are both activated by SFA [28] and highly expressed in the human adipose tissue and functional in adipocytes [29, 30], as well as in the liver [31, 32]. In the present study, mRNA level of TLR2, but not TLR4, was enhanced after TLR4 inhibition in the adipose tissue and the liver. This may be compared to findings in TLR4-KO mice, in which TLR2 over-expression was also observed in the adipose tissue and the liver, and explained why adipose and hepatic low-grade inflammatory response to a long-term high-fat/high sugar diet was maintained in these TLR4-KO mice [33]. Interestingly, in our experiment, the TLR2 overexpression appears to be a very short-term adaptation to TLR4 inhibition, possibly in relation with the postprandial status.

Finally, there was no direct relationship between mRNA levels of PPIR markers (*IL-1β*, *IL-6*, *PAI-1* and *MCP-1*) in the adipose tissue or the liver, 3 h after INH or VEH and their plasma concentrations. In particular, the dramatic differences in mRNA levels between the two groups in the adipose tissue were not reflected at the plasma level, as previously shown in human adipose tissue after a test-meal [13]. Interestingly, this human study was performed in metabolic syndrome patients challenged with a high-fat meal after 12 weeks on a high-fat diet, and showed a postprandial increase in NF-κB activation (p65 expression), mRNA levels of some inflammatory cytokines, and plasma concentration of IL-6. This may suggest a similarity in PPIR in both acute and more chronic high-fat challenge [13]. From the postprandial kinetics of plasma MCP-1, one may suspect an increase in MCP-1 6 h after the meal, although there was a significant difference from baseline but not between INH and VEH at that time. A postprandial increase in MCP-1 in rats pretreated with INH would be consistent with the higher MCP-1 mRNA as measured 3 h after meal in the adipose tissue in this group. However, further specific studies would be required to confirm such a latter phenomena occurring in plasma.

## Conclusion

Taking together, data from the literature and the present study, we propose the following mechanism: the competitive inhibition of TLR4 resulted in an enhanced activation of the TLR2-dependent inflammatory pathways in the postprandial period which, in turn, resulted in a high level of inflammatory activation in the adipose tissue, which may finally contribute to maintain a normal plasma PPIR. In conclusion, we show that TLR4 activation is not specifically required to mediate PPIR, inasmuch as its inhibition led to a similar PPIR in plasma. However, the data indicate that the inhibition of the TLR4 pathway potentiates the TLR2 pathway, resulting in an exacerbated postprandial inflammatory response in the adipose tissue. We propose that TLR2 and TLR4 exert a dual and interdependent mediation of the postprandial inflammatory response, at least in the adipose tissue.

## Abbreviations

Fas: Fatty acid synthase
HFM: High-fat meal
Hprt: Hypoxanthine-guanine phosphoribosyl transferase
ICAM-1: Intercellular adhesion molecule 1
Il-10: Interleukin 10
IL-1β: Interleukin 1β
IL-6: Interleukin 6
INH: Inhibitor
Lpl: Lipoprotein lipase
MCP-1: Monocyte chemoattractant protein-1
PAI-1: Plasminogen activator inhibitor-1
PPIR: Postprandial inflammatory responses
RT-qPCR: Real-time quantitative PCR
SFA: Saturated fatty acid
Tlr: Toll-like receptor
Tnf-α: Tumor necrosis factor α
Vcam-1: Vascular cell adhesion molecule 1
VEH: Vehicle
